# Evaluation of antimicrobial resistance surveillance data sources in primary care setting: a scoping review

**DOI:** 10.1093/fampra/cmaf013

**Published:** 2025-03-28

**Authors:** Vimrata Mori, Gary Grant, Laetitia Hattingh

**Affiliations:** Department of School of Pharmacy and Medical Sciences, Griffith University, 1 Parklands Drive, Southport, Gold Coast, Queensland 4215, Australia; Department of School of Pharmacy and Medical Sciences, Griffith University, 1 Parklands Drive, Southport, Gold Coast, Queensland 4215, Australia; Department of School of Pharmacy and Medical Sciences, Griffith University, 1 Parklands Drive, Southport, Gold Coast, Queensland 4215, Australia; Allied Health Research, Allied Health and Rehabilitation Services, Gold Coast Hospital and Health Services, Gold Coast University Hospital, 1 Hospital Boulevard, Southport, Gold Coast, Queensland 4215, Australia; Department of School of Pharmacy, University of Queensland, UQ Dutton Park, Level 4, 20 Cornwall Street, Woolloongabba, Brisbane, Queensland 4102, Australia

**Keywords:** antimicrobial resistance, health surveillance, resistance data, primary care

## Abstract

**Background:**

Antimicrobial resistance (AMR) is a global health warning that increases mortality, morbidity, and medical expenses. Effective AMR surveillance is essential to guide interventions and maintain treatment efficacy. While AMR surveillance is studied in various healthcare settings, data sources in primary care settings need to be evaluated.

**Aim:**

To identify the value of utilizing AMR surveillance data in primary care settings to inform community antimicrobial stewardship (AMS) practices.

**Methods:**

Eligibility criteria included primary studies, randomized and nonrandomised controlled trials, observational studies, surveys, qualitative studies, mixed-method studies, and grey literature in primary care published worldwide from 2001 to 2024.

**Results:**

Our review of 21 included studies emphasized the significance of utilizing AMR surveillance data to enhance clinical care. Clinicians need to better understand the local AMR pattern when prescribing primary care antibiotics. Despite limitations, educational interventions can change prescribing behaviour. AMR increased because local susceptibility data frequently did not inform empirical antibiotic treatment. Digital and geospatial platforms could enhance surveillance with institutional support and standardized data integration.

**Conclusion:**

This analysis highlights the need for user-friendly, real-time, and easily accessible data visualization platforms to improve AMR surveillance and AMS in primary care. Addressing data accessibility and providing training and education are crucial elements. Standardising data and utilizing digital technologies can improve decision-making and antibiotic prescribing. These elements must be incorporated into a consistent and adaptive plan for effective AMS interventions and public health outcomes.

**Key messages**

- Continuing education on antimicrobial resistance (AMR) education should be further emphasized in medical training and in the professional development of clinicians in primary care to address knowledge gaps.
- Standardising and integrating AMR data at the primary care level will significantly enhance the effectiveness of surveillance efforts.
- Developing a user-friendly, real-time, and easily accessible data visualization platform will enable access to localized AMR data, empowering clinicians to make better-informed decisions.
- Establishing coordinated policy efforts and creating national AMR centres is essential to support robust, adaptable surveillance strategies.
- Building trust in AMR surveillance systems can be accomplished by disseminating localized data and presenting timely feedback to all clinicians and prescribers.

## Introduction

**Figure 1.**
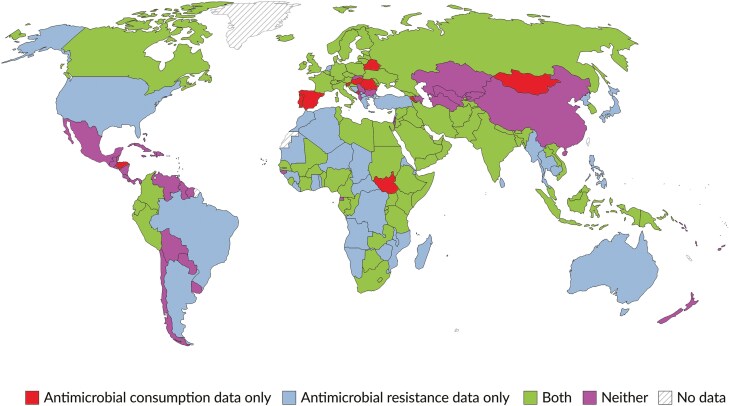
Countries enrolled in the WHO’s GLASS to track data for antimicrobial consumption, resistance, both, or neither, 2024 [5].

The worldwide health issue of antimicrobial resistance (AMR) impacts the health of people, animals, and plants [1]. AMR complicates patient management by limiting the available treatment options, prolonging treatment durations, increasing costs, increasing the chances of being hospitalized, and elevating the danger of serious illness and death [2]. The World Health Organisation developed the Global Antimicrobial Resistance Surveillance System (GLASS) on 22 October 2015 to address this urgent problem [3]. The initiative monitors trends in AMR and promotes responsible antimicrobial usage (AU) worldwide [3]. Through standardized data collection and reporting methodologies, capacity building in resource-limited settings, evidence-based policy development, and fostering international collaboration, GLASS ensures data comparability, strengthens surveillance infrastructure, and encourages the sharing of best practices to combat AMR [3, 4]. As of the most recent data, GLASS includes over 100 participating countries and territories (Fig. 1) [5], covering 65%–70% of the global population [5]. Participating countries contribute data on specific pathogens and AMR trends, compiled into a global report [5]. In addition to GLASS, several large international programmes like the European Antimicrobial Resistance Surveillance Network [6–8], United States National Antimicrobial Resistance Monitoring System [9–11], Asia Pacific Surveillance Network [12, 13], Latin American Network for Antimicrobial Resistance Surveillance [14, 15], and The Fleming Fund [16]. These use high-quality microbiological data and mainly focus on AMR surveillance and monitoring, tracking AMR trends, generating data, and improving surveillance systems in specific regions [6, 9, 12–14, 16].

Consistency in surveillance system data ensures reliability and uniformity across regions and periods. This involves standardized methods and definitions, regular quality checks to verify accuracy and completeness, effective integration of data from various sources and timely data collection and reporting to maintain relevance and actionability [17–19]. Moreover, international collaboration is essential in addressing AMR because it is a complex global issue transcending borders [20]. For several reasons, this approach is more effective than fragmented efforts. First, resistant bacteria can spread worldwide through travel, trade, and migration, making coordinated efforts indispensable [20]. Second, collaborative efforts allow countries to share resources such as funding, expertise, and technology, which is particularly beneficial for low- and middle-income countries (LMICs) [21]. Third, working together helps standardize data collection and reporting methods, enabling effective comparisons and analyses across regions [20]. Some examples of successful international collaboration such include the Joint Initiative on Antimicrobial Resistance [22], the Commonwealth Partnerships for Antimicrobial Stewardship [20, 23], and the Transatlantic Taskforce on Antimicrobial Resistance [24, 25]. These initiatives exemplify how countries and organizations can effectively pool resources and expertise to combat AMR [22–24].

Antimicrobial surveillance systems are pivotal in combating AMR by providing reliable data on resistance patterns, AU, and epidemiological investigations [26–28]. These systems enable the prompt notification of surveillance data to clinicians to facilitate effective treatment and outbreak management strategies [28]. Such systems can function actively or passively and vary in focus and complexity, playing a vital role in informing and improving antimicrobial stewardship (AMS) programmes across all healthcare sectors [6]. Although AMS is well-established in hospital settings, community AMS activities have received less attention and standardization despite > 80% of antibiotic prescriptions being issued in community settings [29–32]. This discrepancy highlights the need for robust data to inform AMS practices in primary care settings [31]. The Australian Government’s National AMR Strategy 2015–2019 underscores the importance of integrated surveillance [33]. The Antimicrobial Use and Resistance in Australia Surveillance System coordinates multiple data streams to deliver an extensive picture of AMR and AU trends in community and hospital settings [1, 34]. This approach demonstrates how surveillance systems can effectively guide AMS practices across health sectors [1].

The purpose of this scoping review is to identify the value of utilizing AMR surveillance data in primary care settings to inform community AMS practices and highlight the critical role of reliable data in addressing the AMR challenges in primary care. To fulfil this aim, this review focussed on 5 objectives. Firstly, to understand and assess the state of AMR surveillance data and identify gaps to improve this strategy. Secondly, to analyse the utilization of AMR surveillance data in informing community-based AMS practices. Thirdly, to evaluate existing data connections and portals to access data sources. Fourthly, to assess barriers and limitations to accessing specific data sources, and lastly, to evaluate the role of education, public awareness, and evaluation of the effectiveness of interventions. This review is timely and significant given the growing impact of AMR on primary care settings and the need for actionable, up-to-date data to support treatment and public health [35].

## Methods

This scoping review followed the Preferred Reporting Items for Systematic Reviews and Meta-analyses (PRISMA) guidelines for Scoping Reviews (PRISMA-ScR) [36].

### Eligibility criteria

This scoping review included primary studies published between 2001 and 2024 on AMR surveillance data sources and AMS in primary care settings internationally. Eligible studies included randomized controlled trials, nonrandomised controlled trials, observational studies, surveys, qualitative studies, mixed-method studies, and grey literature. Exclusion criteria were applied to maintain the review’s focus and relevance. These were studies that focussed solely on hospital settings, secondary studies, animal models, agricultural settings, genome analysis, polymerase chain reaction (PCR), point-of-care (POC), single laboratory studies, case reports, conference abstracts, and non-English publications (Table 1).

**Table 1.** Inclusion and exclusion criteria for studies evaluating antimicrobial resistance surveillance data sources in primary care setting, worldwide (2001–2024).

Criteria	Inclusion	Exclusion
**Population**	Studies conducted in only primary care settings, especially human-focussed and healthcare-centred worldwide, were published between 2001 and 2024.	Studies are based solely on hospital settings, including emergency rooms, inpatient and intensive care units, and hospital-affiliated outpatient settings. Studies using animal models and agricultural settings because they fall outside the review’s emphasis on AMR surveillance in primary care. Studies published outside the 2001–2024 timeframe.
**Intervention**	Primary studies included for this review focussed on AMR surveillance data sources and AMS practices in primary care, including randomized controlled trials, non-randomized controlled trials, observational studies, surveys, qualitative studies, mixed-method studies, and grey literature (e.g. thesis, reports, and unpublished materials).	Secondary studies. Genome analysis, PCR techniques, or POC testing because they fell outside the review’s emphasis on AMR surveillance in primary care.
**Comparator**	–	Single laboratory studies or case reports due to limited generalisability.
**Outcome**	Insights into AMR surveillance data sources and gaps in AMR surveillance data to inform community AMS practices: Understanding and assessing the state of AMR surveillance data. Utilisation of AMR surveillance data. Evaluate existing data connections and portals for access to data sources. Identify barriers and limitations to accessing AMR surveillance data. Assessing the role of education and public awareness and evaluating the effectiveness of interventions.	Conference abstracts and non-English publications to avoid potential data accuracy and consistency.

### Information sources

The primary sources of information were the key term searches and topic headings across all databases (Supplementary Table 1) and electronic databases MEDLINE (Ovid) (Supplementary Table 2), Embase (Supplementary Table 3), and PubMed (Supplementary Table 4). The Search occurred from April to May 2023, and the latest version occurred again in April 2024. Furthermore, back-citations were analysed to support the electronic database search. This involved reviewing the included publications’ reference lists and examining pertinent articles found through AMR surveillance-related searches to ensure they met the review’s eligibility requirements. The PRISMA-ScR checklist can be found in Supplementary Table 5.

### Evidence Source Selection

All retrieved records were imported into Endnote 21 reference management software to eliminate duplicates and initially screen titles and abstracts. All three reviewers (VM, GG, and LH) managed the screening process together, narrowing the records to 100. All three reviewers independently analysed the articles for full-text review and eligibility assessment, and the reviewers’ differences were settled by consensus.

## Results

### Selection of evidence sources

**Figure 2.**
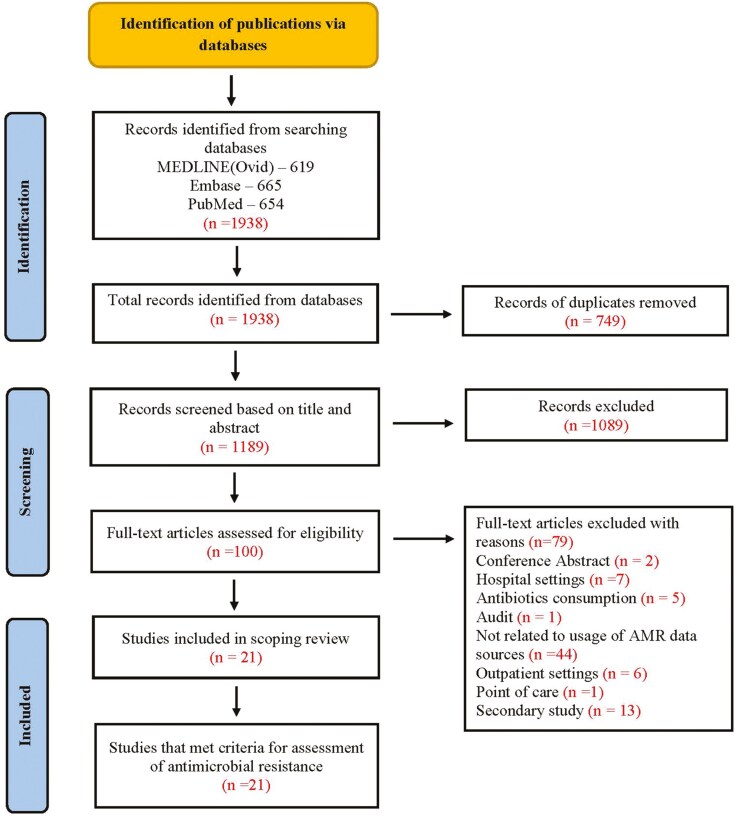
Illustrates the PRISMA-ScR flow diagram (preferred reporting items for systemic reviews and meta-analyses extension for scoping review) [36].

In the initial search, 1938 articles were found. In total, 1189 articles were screened for titles and abstracts after removing 749 duplicate entries. Of these, 1089 articles were excluded because they did not fit within the study scope based on the eligibility criteria. Out of them, 79 were eliminated; based on the exclusion criteria, the reason for this exclusion is given in (Fig. 2), the remaining 21 articles for the review.

### Data extraction

The lead reviewer (VM) designed a data extraction form in Microsoft Excel 365 to identify the critical variables for extraction. After a discussion among all team members, the form was amended through several iterations until a consensus was reached. Eligible articles were then subjected to data extraction (Table 2). The standardized data extraction technique we used included the author, year, reference, country, study aim or purpose, participant kinds and numbers, study type, data type, methodology, and knowledge gaps based on our 5 objectives.

**Table 2.** Evaluation of antimicrobial resistance surveillance data sources in primary care settings: study aims, participants, characteristics, methodologies, and knowledge gaps, worldwide (2001–2024).

Value of utilizing AMR surveillance data in primary care settings									
First Author, Year, and Reference	Country	Aim/Purpose	Participant types and Numbers	Study type, data types, and methodologies	Knowledge gaps based on 5 objectives				
					1	2	3	4	5
Richards [37]	Christchurch (New Zealand)	To determine the resistance rates and patterns in bacteria causing uncomplicated UTIs presenting to general practitioners in Christchurch	82 GPs 374 women (16–50 years)	Primary Quantitative Random Urine samples were collected	✔				
Simpson [38]	UK	To gain a better comprehension of GPs’ perceptions of AMR	40 GPs (14 from regular fluoroquinolone prescribing practices and 26 from high fluoroquinolone prescribing practices)	Primary Qualitative Semi-structured interview		✔			
Catal [39]	Turkey	To determine the alternatives for children with community-acquired UTIs who get empirical antibiotic therapy by evaluating the changing susceptibility of urinary bacteria to routinely used antimicrobials over 6 years	698 paediatric patients’ urine samples (less than 15 years old)	Primary Quantitative Retrospective analysis Study			✔		
Kotwani [40]	India	To research potential interventions and investigate the factors that influence primary care physicians’ prescriptions of antibiotics	36 primary care physicians (3 focussed groups)	Primary Qualitative Focused group discussions		✔		✔	✔
Etienne [41]	France	To investigate rapid urine test -daily use and local epidemiology to identify the optimal strategy	347 females (age 18–65 years)	Primary Quantitative Urine analysis	✔	✔			
Fleming [42]	Ireland	To explore healthcare professionals’ views on antibiotic prescribing in long-term care facilities	37 participants (4 consultants, 10 GPs, 9 pharmacists, and 14 nurses)	Primary Qualitative Semi-structured interviews				✔	✔
Duane [43]	Ireland	To explore the community members’ and GPs’ perspectives on the culture of consumption and prescribing antibiotics in the community for UTI	42 participants (6 focus groups and 15 GPs)	Primary Qualitative In-depth interviews with participants in a focus group				✔	✔
Wilcock [44]	UK	To evaluate GPs’ knowledge of AMR and AMS, how this affects their prescribing choices, and their recommendations for enhancing antibiotic prescribing strategies	45 GPs	Primary Qualitative Questionnaire					✔
Bathoorn [45]	Netherland	To examine adherence to these guidelines regarding antibiotic treatment in acute exacerbations of chronic obstructive pulmonary diseases in primary care and the use of sputum culture	36,172 regional data	Primary Quantitative Retrospective Cohort Study			✔		
Anderson [46]	UK	To develop the intervention, we explored primary care physicians’ perspectives about a locally relevant, real-time viral surveillance system prototype that assists with paediatric respiratory tract infection diagnosis and antibiotic prescription. We investigated the clinicians’ opinions on the system’s content, expected use, and effect	21 participants (18 GPs and 3 nurses)	Primary Qualitative Semi-structured interviews (face-to-face)	✔				✔
Bakhit [47]	Australia	To investigate how patients or parents of children’s patients perceive AMR and resistance-related concepts, such as resistance reversibility and its transmission among nearby individuals, and how this might affect their perceptions of AU for acute respiratory infections	32 child patients or their parents	Primary Qualitative Semi-structured interviews					✔
Al-Haboubi [48]	UK	Prescribers and other interested parties should be notified as soon as possible to enhance the therapy’s suitability for treating resistant diseases, efficiently control outbreaks, and facilitate the development of programmes and tactics to lessen or stop the emergence of new resistance	112 participants (41 national policymakers and 71 HPs)	Primary Qualitative Semi-structured interviews	✔	✔	✔		✔
Dadzie [49]	Ghana	To analyse the Tema Metropolis pneumonia surveillance system’s characteristics and see if it is accomplishing its goals	20 participants (Physicians, community health nurses, laboratory scientists, disease control officers, record personnel, and health information officers)	Primary Mixed method Semi-structured interviews, observations, and records review	✔			✔	
Wozniak [50]	Australia	To create an interactive, geospatial and online platform for gathering, evaluating, and sharing data on regional bacterial pathogen susceptibility	4 pathology laboratories	Primary Quantitative Collection of data from laboratories		✔			
Alradini [51]	Saudi Arabia	To determine the opinions of physicians regarding the antibiotics prescribed in various	434 primary healthcare physicians	Primary Qualitative Cross-sectional the theory of planned behaviour model questionnaire was employed in the Questionnaire-based study				✔	✔
Guo [52]	Singapore	To investigate the processes underlying the prescription of antibiotic prescribing by taking into account physicians’ backgrounds in various primary care settings	30 participants [17 doctors working in publicly funded primary care clinics (polyclinics) and 13 GPs working in private practises (solo, small, and large)]	Primary Qualitative Semi-structured interviews	✔				
Saliba-Gustafsson [53]	Malta	To investigate GPs’ perception of AU and AMR in Malta and discuss the obstacles and enablers they see to cautiously prescribing antibiotics for RTIs	20 GPs	Primary Qualitative Face-to-face individual semi-structured interviews				✔	
Guma [54]	South Africa	To evaluate important variables related to private sector GPs’ empirical antibiotic prescribing in South Africa’s Thekwini area, specifically for patients with ARIs	209 GPs	Primary Qualitative Observational, analytic, cross-sectional model (semi-structured web-based questionnaire)			✔	✔	
Penalva [55]	Spain	To investigate if a quarterly AMR/AMU surveillance system in the EU/EEA area is feasible and scalable	11 European countries	Primary Quantitative Longitudinal study	✔	✔			
Shrestha [32]	Nepal	To identify potential local causes of AMR in humans and drug resistance patterns in *Escherichia Coli (E. coli)* found in healthy adult faecal samples in specific Dhulikhel municipality wards	424 participants (quantitative—above 18 years of age, qualitative (health coordinators, food vendors, pharmacies and community members)	Primary Mixed method, Quantitative (cross-sectional study) Qualitative (focus group discussion)		✔			✔
da Silva-Brandao [56]	Brazil	To investigate how local and multilevel socioeconomic factors affect the prescription and distribution of antibiotics in Brazil’s primary care setting for human and animal health	25 participants (GPs, nurses, dentists, pharmacists, and veterinarians)	Primary Qualitative Semi-structured interviews and contextual observations				✔	✔

### Characteristics of Evidence Sources

The 21 studies were published between 2001 and 2024, with about 15 published after GLASS was implemented on 22 October 2015. Geographically, the studies represented a broad international spectrum. Studies were conducted in various countries (Table 2), including New Zealand (1) [37], UK (4) [38, 44, 46, 48], Turkey (1) [39], India (1) [40], France (1) [41], Ireland (2) [42, 43], Netherland (1) [45], Australia (2) [47, 50], Ghana (1) [49], South Arabia (1) [51], Singapore (1) [52], Malta (1) [53], South Africa (1) [54], Spain (1) [55], Nepal (1) [32] and Brazil (1) [56]. Of the 21 studies, 15 (71.4%) highlighted a notable absence of AMR data within primary health care [32, 37–41, 43, 45, 48–50, 52, 53, 56, 54]. Two (9.5%) studies explored the nuances of antibiotic prescription habits and the level of awareness of AMR [44, 51]. One (4.7%) study focussed on monitoring viral infections in children [46], One (4.7%) was based on the understanding of AU and AMR among patients and their guardians [47], and One (4.7%) investigated the role of local and regional data on respiratory pathogens susceptibility in influencing antibiotic prescription choices [55]. Furthermore, One (4.7%) study concentrated on the behavioural aspects influencing antibiotic prescription in long-term care settings [42]. Topics covered prescription behaviour, patient perspectives, innovative surveillance systems and the need for localized AMR data.

### Synthesis of results

All 21 studies evaluated the value of utilizing AMR surveillance data in primary care, categorized on the 5 objectives (Table 3).

**Table 3.** Studies evaluated AMR surveillance data sources in primary care settings by 5 objectives: gaps, accessibility, and their role in informing AMs practices, worldwide (2001–2024).

References	Aim: To identify the value of utilizing AMR surveillance data in primary care settings to inform community AMS practices	No. of studies out of a total of 21 studies (%)
[37, 41, 46, 48, 49, 52, 55]	**Objective 1**: To understand and assess the state of AMR surveillance data and identify gaps to improve this strategy in primary care settings.	7 (33.3%)
[32, 38, 40, 41, 48, 50, 55]	**Objective 2:** To analyse the utilization of AMR surveillance data in informing community-based AMS practices.	7 (33.3%)
[39, 45, 48, 54]	**Objective 3:** Evaluating existing data connections and portals to access data sources in primary care settings.	4 (19.0%)
[40, 42, 43, 49, 51, 53, 56, 54]	**Objective 4:** To assess barriers and limitations to accessing specific data sources in primary care settings.	8 (38.0%)
[32, 40, 42–44, 46–48, 51, 56]	**Objective 5:** Role of education, public awareness, and evaluation of the effectiveness of interventions.	10 (42.6%)

#### Objective 1: 7 (33.3%) studies focussed on understanding and assessing the state of AMR surveillance data and identifying gaps to improve this strategy in primary care settings [37, 41, 46, 48, 49, 52, 55]

An urgent need was determined to raise awareness about existing data platforms for combating AMR, with an emphasis on enhancing their utility through user-friendly design and providing educational support for clinicians [48]. Clinicians often distrust population-based surveillance data because of cognitive biases, such as anchoring and adjustment, availability, and representativeness biases. These biases have raised concerns about the data’s relevance and applicability to clinical practice. To address this gap, strategies were suggested for presenting data in ways that align with clinicians’ decision-making processes, which are influenced by the “representativeness heuristic” [46]. The existing AMR surveillance system exhibited positive attributes but revealed gaps in laboratory capabilities that could undermine data reliability if not addressed [41]. One study reflected local epidemiological data, countering issues associated with value-based prescription practices [52]. This approach prioritizes data specific to a local population’s health conditions rather than basing prescribing decisions on factors such as drug costs or general effectiveness across a broad population. By tailoring treatments to regional health needs, these guidelines effectively reduced the risks of under- or over-prescribing specific therapies [52]. Another study advised that optimizing and reducing antibiotic prescribing based on recent local epidemiology data for Urinary Tract Infections (UTIs) rather than global data should guide antibiotic prescribing and the need to improve strategies for AMR surveillance in primary care settings [41]. The author emphasized that such tailored guidelines could help clinicians make more informed, contextually relevant decisions [49]. A more frequent, quarterly surveillance system was deemed achievable but would require committed institutional support and integration of multiple data sources [55]. The importance of accurate and timely data from local community laboratories for early warning and clinical guidance has been underscored, calling for an assessment of current laboratory capabilities [37]. Overall, studies highlighted areas for improvement, indicating that enhancing the quality, accessibility, and utilization of AMR surveillance data is pivotal to optimizing its application in primary care settings.

#### Objective 2: 7 (33.3%) studies analysed the utilization of AMR surveillance data in informing community-based AMS practices [32, 38, 40, 41, 48, 50, 55]

The significance of local community laboratory data was highlighted as an early detection system for new resistance and a guide for AMS in clinical practice [48]. Academic detailing for physicians and microbiological data-supported guidelines were found to effectively reduce inappropriate AU, highlighting the need for evidence-based AMS strategies [40]. In the European Union/European Economic Area (EU/EEA), longitudinal research assessed the viability and flexibility of a quarterly AMR/AU surveillance system [55]. A pilot study gathered data on AMR and AU quarterly, and its findings demonstrated that quarterly surveillance provided a helpful tool for the early identification of elevated or decreased AU in the community. The study proposed developing a more robust quarterly surveillance system for AMR and AU [55]. A Study in Nepal supported the value of comprehensive AMR data, supplemented by the global call to assess resistance patterns of *Escherichia coli (E. coli)* at the community level. The results can be used to compare resistance patterns found by regular AMR surveillance at the community level, ultimately assessing the effectiveness of AMS practices over time [32]. The role of microbiologists in identifying local baseline resistance patterns was emphasized. It was recommended that integrating these data with the prescribing histories of clinicians could make the information more actionable [38]. To support this, a study highlighted the use of local epidemiology data for UTIs to reduce inappropriate AU in community-based AMS practices [41]. Furthermore, a multidimensional approach to AMR surveillance that integrates social factors has been indicated to enhance the efficacy of AMS practices [50]. These studies underscored the multifaceted requirements for a robust, evidence-based AMS strategy that effectively utilizes AMR surveillance data in community settings.

#### Objective 3: 4 (19.0 %) studies evaluated existing data connections and portals to access data sources in primary care settings [39, 45, 48, 54]

A 2020 study highlighted that the UK’s current AMR and AU surveillance data systems possessed timeliness, stability, simplicity, and flexibility. However, it lacked a robust laboratory component, underscoring the need for future interventions to address this gap [48]. A study found that Improvements in data connections and portals could give clinicians real-time access to local epidemiological data, facilitating the immediate initiation of empiric antibiotic treatments based on culture results [45]. A study highlighted that more local surveillance data systems could provide clinicians with quick and detailed knowledge of the prevalence of resistance, enabling them to make better-informed decisions about preventive treatments and reduce unnecessary AU [39]. Furthermore, a 2022 study conducted in South Africa highlighted the need for the South African AMR National Strategy Framework to evaluate current systems to ensure prompt delivery of localized data [54]. These studies highlighted the critical need for enhanced data connections and portals to facilitate effective, data-driven AMS practices in primary care settings.

#### Objective 4: 8 (38.0%) studies assessed barriers and limitations to accessing specific data sources in primary care settings [40, 42, 43, 49, 51, 53, 56, 54]

A potential barrier identified was the lack of physician awareness regarding public health issues surrounding AMR, which could limit their motivation to access or comprehend relevant data [51]. The inadequacy of laboratory components was identified as a significant gap in the existing surveillance system, suggesting the need for improvement [49]. The Structured Implicit Mean-field Primal-dual Learning (SIMPLe) programmes were highlighted as a multifaceted approach to overcome barriers, incorporating professional development programmes and electronic prescribing prompts for clinicians [43]. The authors used the Capability, Opportunity, Motivation, and Behaviour (COM-B) model and the Behaviour Change Technique (BCT) Taxonomy to show how focussed educational and feedback mechanisms could have successfully addressed data access barriers [42]. The COM-B model provides a comprehensive structure for assessing behaviour change interventions by determining which components—capability, opportunity, or motivation—must be addressed to promote behaviour change effectively [42]. This method made it possible to create interventions specifically adapted to the needs and situations of researchers and clinicians, increasing the potential for success [57, 58]. The BCT taxonomy provides a systematic method for researchers and clinicians to describe and classify techniques used in behavioural change interventions. This approach promoted transparency in intervention strategies, allowed for comparisons across studies, and informed the development of more effective behavioural change approaches [57–59]. The unintended consequences of guideline availability indicated that the design and accessibility of such resources could limit their effectiveness [54]. Furthermore, the need for data-driven, evidence-based AMS practices was emphasized [40]. Community AMR rates and inadequate access to national guidelines were noted as significant barriers, underscoring the necessity of a strong digital platform to address these gaps [53]. In LMICs, limited healthcare resources and lack of follow-up care lead to over-prescribing of antibiotics. Clinicians often use antibiotics as a safe option due to socioeconomic pressures and perceived patient needs. This finding highlighted the need for better access to local AMR data and targeted educational programmes [56]. Overall, these studies identified the wide range of barriers and limitations that must be addressed to enhance the utility and access of AMR data in primary care settings.

#### Objective 5: 10 (42.6%) studies highlighted the role of education, public awareness, and evaluation of the effectiveness of interventions [32, 40, 42–44, 46–48, 51, 56]

A critical need for targeted educational interventions was identified to address clinicians’ behavioural factors and gaps in knowledge concerning AMR bacteria [48, 51]. One study proposed using educational strategies to overcome clinicians’ reluctance to utilize population-level surveillance data, encouraging more data-driven decision-making [46]. The types of information most effective in public messaging and clinical consultations were identified to enhance educational campaigns [47]. Professional development programmes and audit-feedback mechanisms were identified as essential in breaking down primary care setting barriers by encouraging clinicians to continuously learn and improve their knowledge, abilities, and best practices. This enables them to adapt to evolving healthcare challenges, including the management of complex conditions, effective patient communication, and adherence to treatment guidelines [42, 43]. To encourage rational AU in the community, interventions, including patient awareness campaigns, joint decision-making procedures, continuing medical education (CME) for doctors, and more accurate rules and regulations, have been recommended [40, 60]. The need for patient education to reduce unnecessary antibiotic demand highlighted a significant gap in educational initiatives [32, 44]. Overprescribing in LMICs is also linked to socioeconomic factors and lack of education about AMR. Educational initiatives and public awareness campaigns are needed to improve AU, particularly in vulnerable communities [56]. These studies underscore the importance of multifaceted educational strategies targeting clinicians and the public to ensure more effective AMS practices.

## Discussion

This scoping review evaluated AMR surveillance data in primary care settings. Identified studies demonstrated the value of utilizing AMR surveillance data in primary care settings whilst highlighting the opportunities and challenges of using AMR surveillance data to improve AMS practices. The analysis emphasizes the need for targeted efforts to fill the gaps in data accessibility, availability, and utilization while concentrating on the educational and behavioural aspects of clinicians and the public.

The included studies collectively emphasized that robust AMR surveillance systems are pivotal to the success of AMS programmes. Although existing systems exhibited strengths such as timeliness and simplicity, gaps in laboratory capabilities and data connectivity were consistent limitations [32, 39, 45, 48, 49, 54]. A recurring theme across studies was the need for user-friendly data platforms and educational support to bridge the gap between clinicians and AMR surveillance data, particularly in an era where data-driven decision-making is paramount [48]. A significant barrier identified was clinicians’ distrust of population-based surveillance data, often from cognitive biases and concerns about data relevance [46]. Several studies indicated that enhancing local laboratory capabilities and integrating data from community-level sources could bridge critical gaps [32, 48, 49, 55]. Tailored guidelines based on real-time local epidemiological data effectively address value-based prescribing practices and improve context-specific decision-making in primary care [39, 41, 45, 52]. Another study advocated the necessity of localized data for optimal clinical outcomes. This commonality underscores the need to redesign existing platforms or build new systems that incorporate real-time data requirements [55].

Additionally, the feasibility of implementing a quarterly AMR and AU surveillance system was demonstrated, providing an actionable framework for improving the frequency and granularity of surveillance [55]. Public awareness and lack of knowledge among clinicians may be a potential barrier to adopting AMR surveillance systems [51]. Interventions such as professional development programmes, CME, targeted education, audit-feedback mechanisms, and shared decision-making strategies were found to significantly build trust in AMR surveillance systems and enhance the rational use of antibiotics [40, 42, 43, 56]. The role of patient education in reducing unnecessary antibiotic demand was also underscored, revealing a critical gap in current initiatives [32, 44]. Even with well-designed guidelines in place, their user-friendliness remains a challenge. In primary care settings, the availability of guidelines has occasionally resulted in a rise in the prescribing of empiric antibiotic treatments, likely due to a disconnect between recommendations and practical application. Unlike hospitals, where guidelines are integrated into workflows and supported by stewardship teams, primary care practices often lacks similar resources, leading to an overreliance on empiric prescribing [56, 54]. This highlights the need for a thorough review of how guidelines are formatted, shared, and incorporated into clinical practice to ensure they are both practical and easy to follow.

A comprehensive approach considering clinical, technological, and sociocultural factors is required for effectively addressing AMR. The need to incorporate social determinants into AMR surveillance reminds us that clinical and microbiological data alone are insufficient for a comprehensive understanding of AMR dynamics. Thus, an effective AMR surveillance system would require the concentrated efforts of a diverse set of stakeholders, from microbiologists to public health policymakers, all working collaboratively to create a multidimensional strategy that is both robust and adaptable [50]. Especially in LMICs, clinicians may overprescribe antibiotics, which are influenced by socioeconomic factors like limited healthcare resources, the inability to follow up with patients, and the symbolic value of antibiotics as a quick solution to perceived health risks [56]. This differs from regions with more resources, where healthcare systems might focus more on guidelines and infrastructure to reduce unnecessary prescriptions. Therefore, the challenges faced in AMR surveillance and AMS can vary depending on the level of healthcare resources and socioeconomic conditions in different regions [56].

The results of this study demonstrated the pressing need for a more integrated and comprehensive approach to AMS in primary care. Even though current systems and treatments provide insightful information, the absence of timely, localized, and standardized data remains a significant barrier. Improving laboratory capabilities and integrating data connections across several platforms should be prioritized to ensure real-time access to actionable information. Furthermore, addressing the cognitive biases that hinder clinicians from utilizing surveillance data effectively is crucial. Strategies should include tailored educational programmes and behavioural interventions, such as those guided by the COM-B model and BCT Taxonomy [42, 57]. Such approaches could facilitate more data-driven decision-making by aligning the presentation of AMR with clinicians’ risk-oriented mindsets. The importance of public awareness campaigns cannot be overstated. It is essential to educate patients about the risks of AMR and the significance of careful AU to reduce unnecessary demand. In addition, stricter regulations and shared decision-making frameworks can further support the community AU. This underscores the need to ensure that guidelines are user-friendly, contextually relevant, and supported by tools and training to promote reasonable AU in primary care.

Lastly, more research is needed on integrating social determinants and community-specific factors into AMR surveillance and AMS strategies. This multidimensional approach could enhance the relevance and applicability of AMS interventions and improve their effectiveness in diverse primary care settings.

### Limitations

The limitations of this review should be carefully considered when interpreting the findings. Our inclusion criteria were narrow, focussed on AMR surveillance data in primary care settings and excluded data from hospital settings. The literature search was limited to publications between 2001 and 2024, and we focussed on primary studies, introducing a selection bias. Primary studies often originated from regions or institutions with established AMR surveillance systems and sufficient resources. Consequently, the findings may disproportionately reflect well-resourced settings and countries, while challenges and experiences in the low-resource areas remain underrepresented. This limits the ability to generalize the conclusions to all global contexts. This review may reflect biases in the available literature because regions without AMR surveillance systems or limited resources are less likely to publish studies on AMR surveillance systems.

Pre-print literature was not included, potentially missing the most recent advancements in the field. Our search was restricted to English-language studies, excluding potentially relevant research in other languages. The methodology for sourcing grey literature deviated from traditional systematic approaches, instead utilizing a database specifically chosen for this review and evaluating government and health organization funding carefully considering the researchers past knowledge. These limitations could affect the review’s comprehensiveness and generalisability.

### Conclusion

The studies that are part of this analysis provide a thorough overview of the challenges and opportunities associated with AMR surveillance and AMS practices in primary care. A significant barrier is the lack of a user-friendly, real-time, and easily accessible data visualization platform to guide empirical antibiotic treatment, adversely affecting patient outcomes, and public health. Many clinicians, particularly those in primary care, remain unaware of local AMR patterns because of incomplete, inconsistently maintained, or delayed surveillance systems. This gap compromises the clinician’s ability to make informed decisions and intensifies AMR.

Improving data accessibility and providing targeted training is crucial to bridge these gaps. Fostering education about AMR and AMS is essential because reliance on clinical experience alone is insufficient to address this complex issue. Standardising and integrating data across different levels of health care is equally essential for improving trend identification and resolving inconsistencies in current systems. Advances in digital technologies, such as geospatial surveillance platforms, present promising solutions to enhance data usability and accuracy. Addressing gaps in data systems, promoting education and behaviour change, and raising public awareness are vital steps towards improving AMS in primary care. Future initiatives must focus on incorporating these components into a coherent locally relevant plan and adaptable to the evolving landscape of AMR. By doing so, healthcare systems can implement more effective interventions and optimize antibiotic prescribing practices.

## Supplementary Material

cmaf013_suppl_Supplementary_Tables_1-5

## Data Availability

The data underlying this article are available in the article and in its online supplementary material.
