# Supplementary material for: Evaluation of antimicrobial resistance surveillance data sources in primary care setting: a scoping review
Source: Fam Pract. 2025 Mar 28;42(2):cmaf013. doi: 10.1093/fampra/cmaf013 (PMC11953028; doi:10.1093/fampra/cmaf013)
Supplement: cmaf013_suppl_Supplementary_Tables_1-5 [file cmaf013_suppl_supplementary_tables_1-5.pdf]

Supplementary Table 1: Key Term Search Strategy Detailed Information from 2001 – 2024 worldwide

Sources	Search Terms	Search Dates
MEDLINE(Ovid)	Key term search "Antimicrobial stewardship" or "antimicrobial resistance" or "antibacterial resistance" or "antimicrobial usage" or "antibacterial usage" or "antimicrobial prescribing" or "antibiotic prescribing" or "antibiogram" OR Topic headings Drug resistance, Bacterial OR Drug resistance, Multiple, Bacterial AND<br><br>Key term search "Communit*" or "General practitioner*" or "Family physician*" or "Primary care" OR Topic headings Physicians, family or physicians, primary care OR<br><br>Primary Health Care AND Key term search "Data source*" or "Data usage" or "data connection*" or "data limitation*" or "data availabilit*" or "data sharing" or "information sharing" or "decision making" OR Topic headings<br><br>Information sources, OR Decision making, OR<br><br>Clinical decision-making or Clinical reasoning	1 January 2001 to 24 April 2024
Embase	Key term search "Antimicrobial stewardship" or "antimicrobial resistance" or "antibacterial resistance" or "antimicrobial usage" or "antibacterial usage" or "antimicrobial prescribing" or "antibiotic prescribing" or "antibiogram" OR Topic headings Drug resistance, Bacterial	1 January 2001 to 25 April 2024

	OR<br>Drug resistance, Multiple, Bacterial<br>AND<br>Key term search<br>"Communit*" or "General practitioner*" or "Family physician*" or "Primary care"<br>OR<br>Topic headings<br>Physicians, family or physicians, primary care<br>OR<br>Primary Health Care<br>AND<br>Key term search<br>"Data source*" or "Data usage" or "data connection*" or "data limitation*" or "data availabilit*" or "data sharing" or "information sharing" or "decision making".<br>OR<br>Topic headings<br>Information sources,<br>OR<br>Decision making,<br>OR<br>Clinical decision-making or clinical reasoning	
PubMed	Key term search "Antimicrobial stewardship" or "antimicrobial resistance" or "antibacterial resistance" or "antimicrobial usage" or "antibacterial usage" or "antimicrobial prescribing" or "antibiotic prescribing" or "antibiogram" OR Topic headings Drug resistance, Bacterial OR Drug resistance, Multiple, Bacterial AND Key term search<br>"Communit*" or "General practitioner*" or "Family physician*" or "Primary care" OR Topic headings Physicians, family or physicians, primary care OR<br>Primary Health Care<br>AND Key term search<br>"Data source*" or "Data usage" or "data connection*" or "data limitation*" or "data availabilit*" or "data sharing" or "information	1 January 2001 to 31 March 2024

	sharing" or "decision making".<br>OR<br>Topic headings<br>Information sources,<br>OR<br>Decision making,<br>OR<br>Clinical decision-making or clinical reasoning	

Supplementary Table 2: Search Strategy Medline(OVID)

Set	Search Statement
1.	("Antimicrobial stewardship" or "antimicrobial resistance" or "antibacterial resistance" or "antimicrobial usage" or "antibacterial usage" or "antimicrobial prescribing" or "antibiotic prescribing" or "antibiogram").mp. [mp=title, book title, abstract, original title, name of substance word, subject heading word, floating sub-heading word, keyword heading word, organism supplementary concept word, protocol supplementary concept word, rare disease supplementary concept word, unique identifier, synonyms, population supplementary concept word, anatomy supplementary concept word]
2.	exp Drug Resistance, Bacterial/
3.	exp Drug Resistance, Multiple, Bacterial/
4.	1 or 2 or 3
5.	("Communit*" or "General practitioner*" or "Family physician*" or "Primary care").mp. [mp=title, book title, abstract, original title, name of substance word, subject heading word, floating sub-heading word, keyword heading word, organism supplementary concept word, protocol supplementary concept word, rare disease supplementary concept word, unique identifier, synonyms, population supplementary concept word, anatomy supplementary concept word]
6.	exp Physicians, Family/ or exp Physicians, Primary Care/
7.	exp Primary Health Care/
8.	5 or 6 or 7
9.	("Data source*" or "Data usage" or "data connection*" or "data limitation*" or "data availabilit*" or "data sharing" or "information sharing" or "decision making").mp. [mp=title, book title, abstract, original title, name of substance word, subject heading word, floating sub-heading word, keyword heading word, organism supplementary concept word, protocol supplementary concept word, rare disease supplementary concept word, unique identifier, synonyms, population supplementary concept word, anatomy supplementary concept word]
10.	exp Information Sources/
11.	exp Decision Making/
12.	exp Clinical Reasoning/ or exp Clinical Decision-Making/
13.	9 or 10 or 11 or 12
14.	4 and 8 and 13
15.	limit 14 to yr="2001 - 2024"

Supplementary Table 3: Search Strategy Embase

Search Queries

No.	Query	Results	Date
#15	(('antimicrobial stewardship':ab,ti OR 'antimicrobial resistance':ab,ti OR 'antibacterial resistance':ab,ti OR 'antimicrobial usage':ab,ti OR 'antibacterial usage':ab,ti OR 'antimicrobial prescribing':ab,ti OR 'antibiotic prescribing':ab,ti OR 'antibiogram':ab,ti) OR 'antibiotic resistance'/exp OR 'multidrug resistance'/exp) AND (('communit*':ab,ti OR 'general practitioner*':ab,ti OR 'family physician*':ab,ti OR 'primary care':ab,ti) OR 'general practitioner'/exp OR 'primary health care'/exp) AND (('data source*':ab,ti OR 'data usage':ab,ti OR 'data connection*':ab,ti OR 'data limitation*':ab,ti OR 'data availabilit*':ab,ti OR 'data sharing':ab,ti OR 'information sharing':ab,ti OR 'decision making':ab,ti) OR 'information source'/exp OR 'medical decision making'/exp OR 'clinical decision making'/exp) AND [01-01-2001]/sd NOT [01-04-2024]/sd AND [2001-2024]/py	665	25 Apr 2024
#14	(('antimicrobial stewardship':ab,ti OR 'antimicrobial resistance':ab,ti OR 'antibacterial resistance':ab,ti OR 'antimicrobial usage':ab,ti OR 'antibacterial usage':ab,ti OR 'antimicrobial prescribing':ab,ti OR 'antibiotic prescribing':ab,ti OR 'antibiogram':ab,ti) OR 'antibiotic resistance'/exp OR 'multidrug resistance'/exp) AND (('communit*':ab,ti OR 'general practitioner*':ab,ti OR 'family physician*':ab,ti OR 'primary care':ab,ti) OR 'general practitioner'/exp OR 'primary health care'/exp) AND (('data source*':ab,ti OR 'data usage':ab,ti OR 'data connection*':ab,ti OR 'data limitation*':ab,ti OR 'data availabilit*':ab,ti OR 'data sharing':ab,ti OR 'information sharing':ab,ti OR 'decision making':ab,ti) OR 'information source'/exp OR 'medical decision making'/exp OR 'clinical decision making'/exp)	692	25 Apr 2024
#13	('data source*':ab,ti OR 'data usage':ab,ti OR 'data connection*':ab,ti OR 'data limitation*':ab,ti OR 'data availabilit*':ab,ti OR 'data sharing':ab,ti OR 'information sharing':ab,ti OR 'decision making':ab,ti) OR 'information source'/exp OR 'medical decision making'/exp OR 'clinical decision making'/exp	483291	25 Apr 2024
#12	'clinical decision making'/exp	73457	25 Apr 2024
#11	'medical decision making'/exp	96611	25 Apr 2024
#10	'information source'/exp	3360	25 Apr 2024

#9	'data source*':ab,ti OR 'data usage':ab,ti OR 'data connection*':ab,ti OR 'data limitation*':ab,ti OR 'data availabilit*':ab,ti OR 'data sharing':ab,ti OR 'information sharing':ab,ti OR 'decision making':ab,ti	364658	25 Apr 2024
#8	('communit*':ab,ti OR 'general practitioner*':ab,ti OR 'family physician*':ab,ti OR 'primary care':ab,ti) OR 'general practitioner'/exp OR 'primary health care'/exp	1314132	25 Apr 2024
#7	'primary health care'/exp	216498	25 Apr 2024
#6	'general practitioner'/exp	123773	25 Apr 2024
#5	'communit*':ab,ti OR 'general practitioner*':ab,ti OR 'family physician*':ab,ti OR 'primary care':ab,ti	1204879	25 Apr 2024
#4	('antimicrobial stewardship':ab,ti OR 'antimicrobial resistance':ab,ti OR 'antibacterial resistance':ab,ti OR 'antimicrobial usage':ab,ti OR 'antibacterial usage':ab,ti OR 'antimicrobial prescribing':ab,ti OR 'antibiotic prescribing':ab,ti OR 'antibiogram':ab,ti) OR 'antibiotic resistance'/exp OR 'multidrug resistance'/exp	293288	25 Apr 2024
#3	'multidrug resistance'/exp	58478	25 Apr 2024
#2	'antibiotic resistance'/exp	232104	25 Apr 2024
#1	'antimicrobial stewardship':ab,ti OR 'antimicrobial resistance':ab,ti OR 'antibacterial resistance':ab,ti OR 'antimicrobial usage':ab,ti OR 'antibacterial usage':ab,ti OR 'antimicrobial prescribing':ab,ti OR 'antibiotic prescribing':ab,ti OR 'antibiogram':ab,ti	60984	25 Apr 2024

Supplementary Table 4: Search Strategy PubMed

11 #3 and #6 and #9 from 2001/1/1 - 2024/3/31 (("Antimicrobial stewardship"[Title/Abstract] OR "antimicrobial resistance"[Title/Abstract] OR "antibacterial resistance"[Title/Abstract] OR "antimicrobial usage"[Title/Abstract] OR "antibacterial usage"[Title/Abstract] OR "antimicrobial prescribing"[Title/Abstract] OR "antibiotic prescribing"[Title/Abstract] OR "antibiogram"[Title/Abstract] OR ("drug resistance, multiple, bacterial"[MeSH Terms] OR "drug resistance, bacterial"[MeSH Terms])) AND ("communit*"[Title/Abstract] OR "general practitioner*"[Title/Abstract] OR "family physician*"[Title/Abstract] OR "Primary care"[Title/Abstract] OR ("physicians, primary care"[MeSH Terms] OR "Primary Health Care"[MeSH Terms] OR "physicians, family"[MeSH Terms])) AND ("data source*"[Title/Abstract] OR "Data usage"[Title/Abstract] OR "data connection*"[Title/Abstract] OR "data limitation*"[Title/Abstract] OR "data availabilit*"[Title/Abstract] OR "data sharing"[Title/Abstract] OR "information sharing"[Title/Abstract] OR "Decision Making"[Title/Abstract] OR ("Decision Making"[MeSH Terms] OR "Information Sources"[MeSH Terms] OR "Clinical Reasoning"[MeSH Terms] OR "Clinical Decision-Making"[MeSH Terms]))) AND (2001/1/1:2024/3/31[pdat]) **654** 3:33:45

10 #3 and #6 and #9 ("Antimicrobial stewardship"[Title/Abstract] OR "antimicrobial resistance"[Title/Abstract] OR "antibacterial resistance"[Title/Abstract] OR "antimicrobial usage"[Title/Abstract] OR "antibacterial usage"[Title/Abstract] OR "antimicrobial prescribing"[Title/Abstract] OR "antibiotic prescribing"[Title/Abstract] OR "antibiogram"[Title/Abstract] OR ("drug resistance, multiple, bacterial"[MeSH Terms] OR "drug resistance, bacterial"[MeSH Terms])) AND ("communit*"[Title/Abstract] OR "general practitioner*"[Title/Abstract] OR "family physician*"[Title/Abstract] OR "Primary care"[Title/Abstract] OR ("physicians, primary care"[MeSH Terms] OR "Primary Health Care"[MeSH Terms] OR "physicians, family"[MeSH Terms])) AND ("data source*"[Title/Abstract] OR "Data usage"[Title/Abstract] OR "data connection*"[Title/Abstract] OR "data limitation*"[Title/Abstract] OR "data availabilit*"[Title/Abstract] OR "data sharing"[Title/Abstract] OR "information sharing"[Title/Abstract] OR "Decision Making"[Title/Abstract] OR ("Decision Making"[MeSH Terms] OR "Information Sources"[MeSH Terms] OR "Clinical Reasoning"[MeSH Terms] OR "Clinical Decision-Making"[MeSH Terms])) 672 3:33:07

9 #7 or #8 "data source*"[Title/Abstract] OR "Data usage"[Title/Abstract] OR "data connection*"[Title/Abstract] OR "data limitation*"[Title/Abstract] OR "data availabilit*"[Title/Abstract] OR "data sharing"[Title/Abstract] OR "information sharing"[Title/Abstract] OR "Decision Making"[Title/Abstract] OR "Decision Making"[MeSH Terms] OR "Information Sources"[MeSH Terms] OR "Clinical Reasoning"[MeSH Terms] OR "Clinical Decision-Making"[MeSH Terms] 1,849,142 3:12:40

8 ((("Decision Making"[Mesh]) OR "Information Sources"[Mesh]) OR "Clinical Reasoning"[Mesh]) OR "Clinical Decision-Making"[Mesh] Most Recent "Decision Making"[MeSH Terms] OR "Information Sources"[MeSH Terms] OR "Clinical Reasoning"[MeSH Terms] OR "Clinical Decision-Making"[MeSH Terms] 1,627,536 3:11:45

7 "Data source*"[Title/Abstract] OR "Data usage"[Title/Abstract] OR "data connection*"[Title/Abstract] OR "data limitation*"[Title/Abstract] OR "data availabilit*"[Title/Abstract] OR "data sharing"[Title/Abstract] OR "information

sharing"[Title/Abstract] OR "decision making"[Title/Abstract] "data source*"[Title/Abstract] OR "Data usage"[Title/Abstract] OR "data connection*"[Title/Abstract] OR "data limitation*"[Title/Abstract] OR "data availabilit*"[Title/Abstract] OR "data sharing"[Title/Abstract] OR "information sharing"[Title/Abstract] OR "decision making"[Title/Abstract] 288,226 3:09:53

6 #4 or #5 "communit*"[Title/Abstract] OR "general practitioner*"[Title/Abstract] OR "family physician*"[Title/Abstract] OR "Primary care"[Title/Abstract] OR "physicians, primary care"[MeSH Terms] OR "Primary Health Care"[MeSH Terms] OR "physicians, family"[MeSH Terms] 1,099,274 2:53:06

5 (("Physicians, Primary Care"[Mesh]) OR "Primary Health Care"[Mesh]) OR "Physicians, Family"[Mesh] Most Recent "physicians, primary care"[MeSH Terms] OR "Primary Health Care"[MeSH Terms] OR "physicians, family"[MeSH Terms] 213,8762:52:25

4 "Communit*"[Title/Abstract] OR "General practitioner*"[Title/Abstract] OR "Family physician*"[Title/Abstract] OR "Primary care"[Title/Abstract] "communit*"[Title/Abstract] OR "general practitioner*"[Title/Abstract] OR "family physician*"[Title/Abstract] OR "Primary care"[Title/Abstract] 975,2502:50:30

3 #1 or #2 "Antimicrobial stewardship"[Title/Abstract] OR "antimicrobial resistance"[Title/Abstract] OR "antibacterial resistance"[Title/Abstract] OR "antimicrobial usage"[Title/Abstract] OR "antibacterial usage"[Title/Abstract] OR "antimicrobial prescribing"[Title/Abstract] OR "antibiotic prescribing"[Title/Abstract] OR "antibiogram"[Title/Abstract] OR "drug resistance, multiple, bacterial"[MeSH Terms] OR "drug resistance, bacterial"[MeSH Terms] 140,895 2:48:26

2 ("Drug Resistance, Multiple, Bacterial"[Mesh]) OR "Drug Resistance, Bacterial"[Mesh] Most Recent "drug resistance, multiple, bacterial"[MeSH Terms] OR "drug resistance, bacterial"[MeSH Terms] 104,3572:47:26

1 "Antimicrobial stewardship"[Title/Abstract] OR "antimicrobial resistance"[Title/Abstract] OR "antibacterial resistance"[Title/Abstract] OR "antimicrobial usage"[Title/Abstract] OR "antibacterial usage"[Title/Abstract] OR "antimicrobial prescribing"[Title/Abstract] OR "antibiotic prescribing"[Title/Abstract] OR "antibiogram"[Title/Abstract] "Antimicrobial stewardship"[Title/Abstract] OR "antimicrobial resistance"[Title/Abstract] OR "antibacterial resistance"[Title/Abstract] OR "antimicrobial usage"[Title/Abstract] OR "antibacterial usage"[Title/Abstract] OR "antimicrobial prescribing"[Title/Abstract] OR "antibiotic prescribing"[Title/Abstract] OR "antibiogram"[Title/Abstract] 54,156 2:40:13

Supplementary Table 5: Preferred Reporting Items for Systematic reviews and Meta-Analyses extension for Scoping Reviews (PRISMA-ScR) Checklist

SECTION	ITEM	PRISMA-ScR CHECKLIST ITEM	REPORTED ON PAGE #
**TITLE**			
Title	1	Identify the report as a scoping review.	1
**ABSTRACT**			
Structured summary	2	Provide a structured summary that includes (as applicable): background, objectives, eligibility criteria, sources of evidence, charting methods, results, and conclusions that relate to the review questions and objectives.	2
**INTRODUCTION**			
Rationale	3	Describe the rationale for the review in the context of what is already known. Explain why the review questions/objectives lend themselves to a scoping review approach.	4
Objectives	4	Provide an explicit statement of the questions and objectives being addressed with reference to their key elements (e.g., population or participants, concepts, and context) or other relevant key elements used to conceptualize the review questions and/or objectives.	6
**METHODS**			
Protocol and registration	5	Indicate whether a review protocol exists; state if and where it can be accessed (e.g., a Web address); and if available, provide registration information, including the registration number.	6
Eligibility criteria	6	Specify characteristics of the sources of evidence used as eligibility criteria (e.g., years considered, language, and publication status), and provide a rationale.	6
Information sources*	7	Describe all information sources in the search (e.g., databases with dates of coverage and contact with authors to identify additional sources), as well as the date the most recent search was executed.	7
Search	8	Present the full electronic search strategy for at least 1 database, including any limits used, such that it could be repeated.	7
Selection of sources of evidence†	9	State the process for selecting sources of evidence (i.e., screening and eligibility) included in the scoping review.	7
Data charting process‡	10	Describe the methods of charting data from the included sources of evidence (e.g., calibrated forms or forms that have been tested by the team before their use, and whether data charting was done independently or in duplicate) and any processes for obtaining and confirming data from investigators.	27-31
Data items	11	List and define all variables for which data were sought and any assumptions and simplifications made.	27-31
Critical appraisal of individual sources of evidence§	12	If done, provide a rationale for conducting a critical appraisal of included sources of evidence; describe the methods used and how this information was used in any data synthesis (if appropriate).	Not done
Synthesis of results	13	Describe the methods of handling and summarizing the data that were charted.	9 and Table 2

SECTION	ITEM	PRISMA-ScR CHECKLIST ITEM	REPORTED ON PAGE #
**RESULTS**			
Selection of sources of evidence	14	Give numbers of sources of evidence screened, assessed for eligibility, and included in the review, with reasons for exclusions at each stage, ideally using a flow diagram.	8
Characteristics of sources of evidence	15	For each source of evidence, present characteristics for which data were charted and provide the citations.	8
Critical appraisal within sources of evidence	16	If done, present data on critical appraisal of included sources of evidence (see item 12).	Not done
Results of individual sources of evidence	17	For each included source of evidence, present the relevant data that were charted that relate to the review questions and objectives.	27-32 (Table 1)
Synthesis of results	18	Summarize and/or present the charting results as they relate to the review questions and objectives.	9
**DISCUSSION**			
Summary of evidence	19	Summarize the main results (including an overview of concepts, themes, and types of evidence available), link to the review questions and objectives, and consider the relevance to key groups.	14
Limitations	20	Discuss the limitations of the scoping review process.	17
Conclusions	21	Provide a general interpretation of the results with respect to the review questions and objectives, as well as potential implications and/or next steps.	18
**FUNDING**			
Funding	22	Describe sources of funding for the included sources of evidence, as well as sources of funding for the scoping review. Describe the role of the funders of the scoping review.	Not applicable

JBI = Joanna Briggs Institute; PRISMA-ScR = Preferred Reporting Items for Systematic reviews and Meta-Analyses extension for Scoping Reviews.

* Where *sources of evidence* (see second footnote) are compiled from, such as bibliographic databases, social media platforms, and Web sites.

† A more inclusive/heterogeneous term used to account for the different types of evidence or data sources (e.g., quantitative and/or qualitative research, expert opinion, and policy documents) that may be eligible in a scoping review as opposed to only studies. This is not to be confused with *information sources* (see first footnote).

‡ The frameworks by Arksey and O'Malley (6) and Levac and colleagues (7) and the JBI guidance (4, 5) refer to the process of data extraction in a scoping review as data charting.

§ The process of systematically examining research evidence to assess its validity, results, and relevance before using it to inform a decision. This term is used for items 12 and 19 instead of "risk of bias" (which is more applicable to systematic reviews of interventions) to include and acknowledge the various sources of evidence that may be used in a scoping review (e.g., quantitative and/or qualitative research, expert opinion, and policy document).

From: Tricco AC, Lillie E, Zarin W, O'Brien KK, Colquhoun H, Levac D, et al. PRISMA Extension for Scoping Reviews (PRISMA-ScR): Checklist and Explanation. Ann Intern Med. ;169:467–473. doi: 10.7326/M18-0850
